# Modern Developments in Transcranial Magnetic Stimulation: The Editorial

**DOI:** 10.3390/brainsci12050628

**Published:** 2022-05-11

**Authors:** Nico Sollmann, Petro Julkunen

**Affiliations:** 1 Department of Diagnostic and Interventional Radiology, University Hospital Ulm, Albert-Einstein-Allee 23, 89081 Ulm, Germany; 2 Department of Diagnostic and Interventional Neuroradiology, School of Medicine, Klinikum rechts der Isar, Technical University of Munich, Ismaninger Str. 22, 81675 Munich, Germany; 3 TUM-Neuroimaging Center, Klinikum rechts der Isar, Technical University of Munich, 81675 Munich, Germany; 4 Department of Clinical Neurophysiology, Kuopio University Hospital, P.O. Box 100, 70029 KYS Kuopio, Finland; petro.julkunen@kuh.fi; 5 Department of Applied Physics, University of Eastern Finland, 70210 Kuopio, Finland

Transcranial magnetic stimulation (TMS) is being increasingly applied in neuroscience and the clinical setup [1,2,3,4,5]. Applications of TMS are focused on treatment and diagnostics. Modern advances include, but are not limited to, the combination of TMS with precise neuronavigation, as well as the integration of TMS into a multimodal environment, mainly by guiding TMS applications using complementary techniques such as functional magnetic resonance imaging (fMRI), electroencephalography (EEG), diffusion tensor imaging (DTI), or magnetoencephalography (MEG). The impact of stimulation can be identified and characterized by such multimodal approaches, thus helping to shed light on basic neurophysiology and TMS effects in the human brain [6,7,8,9]. 

This Special Issue entitled “Modern Developments in Transcranial Magnetic Stimulation (TMS)–Applications and Perspectives in Clinical Neuroscience” in *Brain Sciences* received studies covering various applications of TMS, with focuses on neuronavigated TMS (nTMS) for mapping of cortical functions [10,11,12,13,14], treatment and modulatory effects [15,16,17,18,19], and basic neuromechanisms [20,21,22], all clinically relevant and supporting the aims set for the Special Issue. 

Sollmann et al. comprehensively reviewed nTMS motor mapping in clinical application with accompanying validating evidence, and provided additional and new evidence on the parametric prerequisites crucial to ensuring feasible mapping accuracy [13]. In addition, the accompanied multimodal information used (e.g., for fiber tracking of the descending motor tracts) was concluded to provide potential aid and improvements in nTMS applicability, particularly in the critical cohort of patients harboring motor-eloquent brain tumors [13]. Highlighted applications of nTMS in clinical motor mapping include, in addition to the conventionally acknowledged imaging information, the mode of risk stratification and prediction of potential surgical outcomes, as well as observations of neural plasticity related to adjustment and relocation of motor functions within the brains of such patients [13]. Sollmann et al. also highlighted the role of methodological integration into clinical routines and accompanied systems for achieving the full potential of nTMS, while considering that successful application requires comprehensive knowledge of the application and its methodological constraints [13]. The authors see great potential in nTMS and its multimodal applications, not only as a surgical planning tool, but also for providing longitudinal information applicable to prognostics and follow-up examinations [13].

Applying motor mapping in critically ill patients, Schramm et al. demonstrated the use of nTMS as a safe and reliable method for motor mapping in the intensive care unit (ICU) setting, and outlined its possible benefits [12]. The authors demonstrated that in the ICU environment, where imaging with computed tomography (CT) is more applicable than magnetic resonance imaging (MRI) that is conventionally used with nTMS, the post-processed CT images provided a feasible alternative to MRI for neuronavigation [12]. The ICU environment is notoriously challenging, given that electromyography (EMG) for motor mapping with nTMS is highly sensitive to noise coupled to weak signals. The noise in the measured EMG signal has been successfully reduced to a feasible level [12]. Schramm et al. considered the potential applications in the ICU to involve prognostics and monitoring of certain transient complications related to the nTMS motor mapping procedure [12].

By mapping language-related areas, Zhang et al. provided evidence for structural differences on cortical and subcortical levels between language-positive (i.e., locations where upon stimulation, a modulatory effect on language performance was detected) and language-negative areas (i.e., locations where no effect of nTMS was detected) during nTMS language mapping among patients with language-eloquent brain tumors [14]. Their results provided additional and new evidence in patients with glioblastoma multiforme regarding the connection of speech and language function and brain anatomy, with nTMS demonstrating that responsiveness to stimulation is critically related to cortical and subcortical interplay and the rate of speech impairment [14]. They considered that the results further increase confidence in nTMS language mapping and nTMS-based tractography in the clinical setting [14]. In their study, Baro et al. reported a case of nTMS-based tractography application in neurosurgery in a bilingual patient affected by a brain tumor in the left temporal lobe, who underwent nTMS mapping for both languages (Romanian and Italian) [10]. This procedure was considered to disclose the true eloquence of the anterior part of the lesion in both language-related tests [10]. The outcome was verified after surgery, with language abilities remaining intact in both languages [10]. To further develop the protocol of language mapping with nTMS, Ohlerth et al. compared a conventional noun-naming task to an action-naming task, and reported that action naming may be more favorable in nTMS mapping in terms of error rates and may hence improve the accuracy of nTMS-aided preoperative planning [11]. Their findings may have distinct impact on nTMS language mapping routines in clinical setups, where an object-naming task is routinely used despite limited specificity. 

In their study, Phipps et al. reviewed the current heterogeneous literature-based evidence for using repetitive TMS (rTMS) to enhance or restore memory, e.g., for applications in Alzheimer’s disease treatment, and offered several recommendations for the design of future investigations using rTMS to modulate human memory performance [17]. Regarding the potential analgesic effects of rTMS for treatment of chronic refractory pain, Freigang et al. compared two treatment targets and sequences against a sham setting in multi-session therapy for lower back pain, and found indications that treatment on the left dorsolateral prefrontal cortex with 5-Hz rTMS may induce greater pain and stress relief than treatment on the primary motor cortex (M1) with 20 Hz [16]. In addition, Xu et al. provided evidence that an intermittent theta-burst stimulation (TBS) protocol on the ipsilesional M1 could induce immediate neural activity and functional connectivity changes in motor, language, and other brain regions in patients with post-stroke aphasia as observed through fMRI, which could promote functional recovery [18]. In an experimental setting in rats, to shed light on the efficacy of rTMS in multiple sclerosis (MS) treatment, Dragić et al. reported that continuous TBS counteracted with experimental autoimmune encephalomyelitis (EAE)-induced effects on adenosine signaling [19]. Furthermore, it attenuated the reactive state of microglia and astrocytes, thus suggesting a potential TBS-induced reduction in the neuroinflammatory process known to be related to MS [19]. 

In their exploratory study combining EEG and rTMS modulation, Casanova et al. found that in autism spectrum disorder (ASD) subjects, visually evoked and induced gamma oscillations were evident at higher magnitudes of gamma oscillations before rTMS modulation than in neurotypical controls [15]. Recordings after rTMS treatment in ASD revealed a significant reduction in gamma responses to task-irrelevant stimuli, and participants made fewer errors after rTMS neuromodulation [15]. In addition, behavioral questionnaires conducted after treatment revealed decreased irritability, hyperactivity, and repetitive behavior scores [15].

Guerra et al. provide a status update and summarizing review on previously reported findings regarding the potential contribution of TMS in combination with EEG to the understanding of the mechanisms underlying normal brain aging [20]. Continuing with combined TMS and EEG, and to demonstrate the functional phase-dependent relationships between frontal, parietal, and occipital areas of the brain to support EEG-state-dependent TMS, Tabarelli et al. studied a large open dataset [22]. They found a consistent connectivity between parietal and prefrontal regions, whereas occipito-prefrontal connectivity was less marked and occipito-parietal connectivity was comparatively low [22]. The authors consider their results a relevant add-on feature for individualized brain-state-dependent TMS, with possible contributions to personalized therapeutic nTMS applications [22]. 

Kariminezhad et al. reported that the individual paired associate stimulation (PAS) response, whether expressed as long-term depression (LTD)-like or long-term potentiation (LTP)-like effects on motor-evoked potentials (MEPs), were related to the individual repetition suppression (RS) responses observed in the MEP amplitudes [21]. Kariminezhad et al. considered this finding a promising step for predicting TMS neuromodulation outcome based on the individual RS effect [21].

Overall, the original articles, reviews, and case studies included in this Special Issue provide interesting reading, solid evidence, important indicative findings, and summarizing conclusions based on recent literature, hence being of great interest for all those working with clinical applications of nTMS or neuroscience research. We would like to thank the authors for their contributions and wish the readers happy and fruitful reading.
